# Direct microvascular monitoring of a free autologous jejunal flap using microendoscopy: a case report

**DOI:** 10.1186/1472-6815-6-14

**Published:** 2006-09-29

**Authors:** Tahwinder Upile, Waseem Jerjes, Mohammed El Maaytah, Colin Hopper, Adam Searle, Anthony Wright

**Affiliations:** 1 Head & Neck Unit, University College London Hospitals, UK; 2 Unit of Oral & Maxillofacial Surgery, Division of Maxillofacial, Diagnostic, Medical and Surgical Sciences, Eastman Dental Institute & University College London Hospitals, UK; 3 Royal Marsden Hospital, London, UK

## Abstract

**Background:**

Early identification of flap failure is an indispensable prerequisite for flap salvage. Although many technical developments of free flap monitoring have now reached clinical application, very few are considered to be reliable and non-invasive for early recognition of flap failure.

**Case presentation:**

We used microendoscopic technique for microvascular monitoring of free autologous jejunal flap by the direct visualisation of the flow of erythrocytes through the capillary vasculature on both the mucosal and serosal surfaces.

Blood flow was seen to be pulsatile, with individual erythrocytes visible in the capillaries. The best view was obtained when the scope was focussed directly on the capillary rather than the graft surface. The view of the unstained mucosal surface was bland apart from the fine capillary loops which were seen to fill with each pulsatile event. The microendoscopic examination of the serosal surface revealed much larger calibre vessels with obvious blood flow.

**Conclusion:**

The microendoscopic monitoring technique is simple and safe with direct visualisation of blood flow. The technique may also be useful for the monitoring of other free bowel transplants.

## Background

Tissue oxygenation and maintenance of microvascular blood flow in grafted tissues are crucial for flap viability. Early identification of flap failure is a precondition of flap salvage and therefore important for flap prognosis [1]. Although many technical developments of free flap monitoring have now reached clinical application, very few are considered to be reliable and non-invasive for early recognition of flap failure [2]. Therefore, reliable monitoring of free microvascular tissue flaps would be a valuable new tool for clinicians [3,4].

Non-invasive monitoring techniques are procedures of little or no morbidity that may be repeated frequently to assess tissue viability. The ideal non-invasive technique would be safe, sensitive, reliable, reproducible, simple to use and inexpensive [5]. Furthermore, postoperative monitoring of the perfusion of a free flap used in head and neck reconstruction is vitally important in achieving a favourable outcome [6].

Several techniques have now an established place in free flap monitoring, some of them being technically demanding (Microdialysis, pH-measurement, Green light photo-plethysmography monitoring, Hydrogen Clearence-techniques), some being less so (simple clinical observation with registration of flap temperature, colour or laser Doppler ultrasound), but all have their place in free flap monitoring. The most significant non-invasive technique to date being simultaneous Laser-Doppler flowmetry and tissue spectrophotometry which enables early recognition of flap failure [4,7,8].

Free jejunal autografts have been widely used to reconstruct circumferential pharyngeal defects in a single stage [6] with reports of current free jejunal autografts survival rates of 94% or greater, free jejunal autografts salvage rates of up to 100%, and perioperative mortality of 6% or less [9]. Unfortunately, this graft type is associated with some morbidity; however, the procedure is well tolerated, and swallowing function is restored in 80% or more of patients within an average of 9 to 12 days after surgery.

Close postoperative monitoring of the perfusion of the free jejunal autograft is stated to be extremely important in achieving a successful outcome [6]. Many monitoring strategies have attempted to detect early postoperative free flap ischemia in an effort to permit intervention and flap salvage. To date unfortunately no method has achieved widespread clinical acceptance [9].

Previously reported methods of microvascular monitoring have used surrogates of blood flow through the vasculature of the transplant. We report the first report of microendoscopic technique for microvascular monitoring of free autologous jejunal flaps by the direct visualisation of the flow of erythrocytes through the capillary vasculature on both the mucosal and intra-operative serosal surfaces.

## Case presentation

A 30-year-old Middle Eastern male with a history of multiple surgical interventions in the gastro-intestinal tract following carcinoma was chosen to be the subject of this preliminary report. The patient initially underwent colonic transposition as a reconstruction followed by a delayed jejunal transposition due to the development of a 12 cm upper oesophageal corrosive stricture.

History and clinical examination revealed no history of further carcinoma; upper endoscopy and radiological examination revealed residual 8 cm upper oesophageal stricture and right recurrent laryngeal palsy.

### Surgical procedure

The surgical access was through a previous pharyngectomy scar in the line of the right sternomastoid muscle with the raising of sub-platysmal flaps. Dissection continued to reveal the thyrocervical trunk and inferior thyroid artery with the excision of the previously fibrosed jejunal graft and the preparation of good vascular supply to the proximal jejunum. During which a generous cuff of muscosa was preserved with good exposure of the right piriform fossa and the remaining 8 cm gap from the pirifom fossa to the oesophagus.

An upper midline laparotomy was performed with local adhesionolysis. The second loop of the jejunum was identified and harvested on good arcade. The pedicle was ligated and remaining jejunal continiuity established and abdomen closed.

The free flap was transposed to the neck with posterior serosal layers to the proximal and distal ends in an iso-peristaltic fashion. A micro-vascular technique was used to create an end to end arterial anastomosis to the transverse cervical artery and an end to side venous anastomosis to the internal jugular vein.

Once good flow was confirmed (by microendoscopy) with satisfactory peristalsis and mucosal reaction the flap was further inset with proximal and distal fish mouthing techniques

### The microendoscope

An operating room Storz Hopkins II Forward oblique 30° microendoscope (4 mm diameter and 18 cm long) attached via a stroz fibre optic cable was connected to a three chip "Olympus" camera and video monitor. Photographs were taken using a "Sony" video photo-printer.

**Figure 1:**
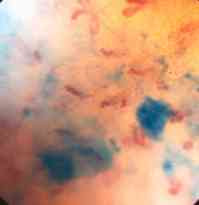
Microendoscopic view ×60 showing methylene blue dye and underlying capillary network at focal length λ of the mucosal surface. The capillary loops are contained within the villi of the jejunum projecting into the lumen hence there tips are visible.

**Figure 2:**
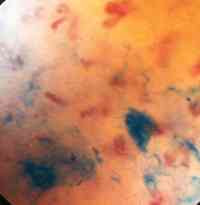
Microendoscopic view ×60 showing methylene blue dye and underlying capillary network at focal length λ of the mucosal surface. At this slightly increased depth of field the projection and part of the base of the villous capillaries are seen.

After suction clearance, the tip of the micro-endoscope was firmly applied to the area of interest to obtain an occlusive contact and then moved for dynamic assessment of the graft's vasculature and blood flow. Both the mucosal and serosal surfaces were examined at 10 randomly chosen sites to show qualitative blood flow in the graft. The examination was reproducible between operators. The depth of field was altered on the microendoscope to visualize the sub mucosal (Figure 1) and sub serosal capillary networks (Figure 2). Further direct microendoscopic monitoring of the mucosal surface was possible in the postoperative period.

**Figure 3:**
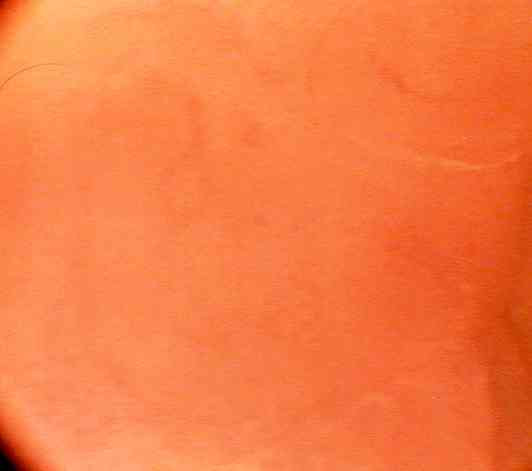
Microendoscopic view ×150 showing mucosal surface and underlying capillary network at time "0". No methylene blue stain was used hence the bland background appearance. The small calibre villous capillaries are barely visible.

**Figure 4:**
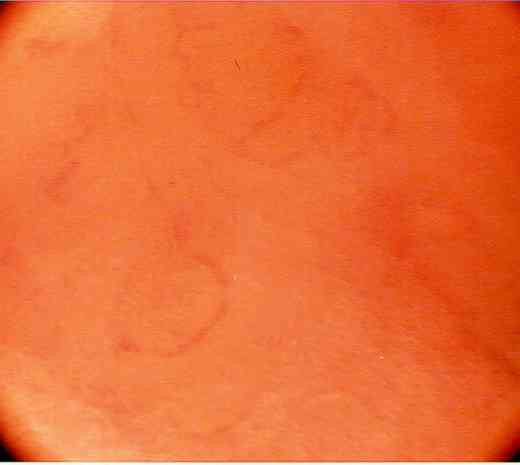
Microendoscopic view ×150 showing mucosal surface and underlying capillary network at time "1". No methylene blue stain was used. Here the capillaries are seen to open up and become visible; this is a pulsatile phenomenon in real time. This suggests good flow and perfusion pressure. The procedure may be done intraoperatively and postoperatively via an endoscope.

**Figure 5:**
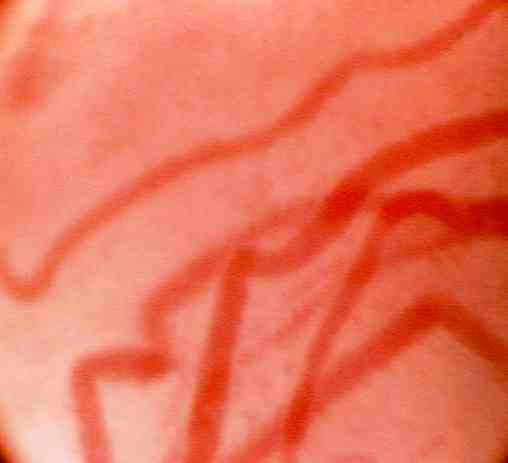
Microendoscopic view ×150 showing serosal surface and underlying capillary network. No methylene blue stain was used. The larger calibre serosal capillaries are easier to visualise, in real time actual cells and flow is visible within the lumen of the vessels. The procedure may be undertaken intraoperatively (during graft setting and before tissue coverage) and on sentinel graft islands postoperatively to assess perfusion. Again time gated image analysis can be performed to provide quantifiable data on blood flow and perfusion with colorimetric analysis possible to assess state of oxygen saturation.

Using this method we were able to directly visualise the flow of blood through the sub-mucosal capillary networks of the transferred free jejunal graft (Figures 3 and 4). This was further confirmed by direct intra-operative visualisation of the blood flow through the vasculature of the graft's serosal surface (Figure 5). This method was used successfully to assess the viability of the flap.

Blood flow was seen to be pulsatile, with individual erythrocytes visible in the capillaries. The best view was obtained when the scope was focussed directly on the capillary rather than the graft surface (Figures 1 and 2). The view of the unstained mucosal surface was bland apart from the fine capillary loops which were seen to fill with each pulsatile event; two consecutive stills were taken to show the capillary loops filling with blood (Figure 3 and 4). Approximately three capillary loops were visible per high power view (×150). Between each pulse of blood flow the mucosal capillary loops were seen to collapse and reduce in visibility.

The microendoscopic examination of the serosal surface revealed much larger calibre vessels with obvious blood flow (Figure 5). The serosal vessels were much easier to visualise and did not collapse between pulses although blood flow through them was seen to be pulsatile.

Microendoscopy was used intraoperatively in a few seconds to assess actual small capillary blood flow in addition to direct observation of the pedicle and edge bleeding. The flow of the erythrocytes can be observed and a time gated image analysis may allow an objective assessment of blood flow. The observations can occur in a real-time and continuous manner during the operation. Perhaps later differentiation of arterial or venous dominant occlusion may be possible through the assessment of oxygen carriage through flow colorimetry. Poor persistent blood flow may be an indication for early surgical intervention.

## Conclusion

Early diagnosis of flap failure is a precondition for flap salvage [1,4,10]. Regardless of the surgeon's experience, thrombosis of the microvasculature is a potential complication depending on several incalculable factors of each individual patient. Thus, postoperative monitoring of autologous microvascular transplants is an absolute necessity to further increase the success rate of this procedure. On one hand, the time interval needed for re-establishing vascular flow is the decisive factor for a successful revision. On the other hand, the certainty about the viability of a flap prevents too early or unnecessary revision [11,4]. Clinical observation is still the normal standard for free tissue transfer monitoring, but continuous observation by the surgeon is not possible, is subjective and depends heavily on experience [4].

Most previously reported microvascular monitoring techniques have been used as surrogates of blood flow through the vasculature of the transplant or have used visible serosal islands through a surgical window with external measuring devices [12,13].

In this preliminary report, we present the non-invasive application of microendoscopic technique for microvascular monitoring of a free autologous jejunal flap by the direct visualisation of the flow of erythrocytes through the capillary vasculature on both the mucosal and intra-operative serosal surfaces. The microendoscopic monitoring technique is simple and safe with direct visualisation of blood flow. The technique may also be useful for the monitoring of other free bowel transplants.

Intraoperatively this novel technique of mucosal and serosal micro-endoscopic assessment allows one to check the viability of the graft micro-vasculature, allowing one to further optimise the vascular anastomosis if found to be inadequate. In cases of sluggish or poor flow the technique may allow early prediction of failure even after restoration of mucosal continuity by serosal monitoring.

Postoperatively, the technique may allow further graft monitoring as a more sensitive adjunct to clinical monitoring to detect early graft failure. This method can be used in accessible sites to view the mucosal surface of the graft; the technique may be modified to utilise a surgical window on the anterior cervical flap and directly visualise the blood flow through the serosal capillaries.

The potential pitfall of the technique is that it is a new skill and the equipment is still relatively expensive. Postoperative access for assessment of the mucosa of inter-positional free grafts for lower pharyngeal reconstructions is restricted and may necessitate further endoscopy, although this may be overcome by serosal island monitoring. We are currently undertaking a prospective study to further assess the potential benefits of this new technique of monitoring in microvascular free transfers.

## Competing interests

The author(s) declare that they have no competing interests.

## Authors' contributions

Upile T: designed the study, carried out the literature research, clinical study and manuscript preparation.

Jerjes W: carried out the literature research, manuscript preparation, and manuscript review.

El Maaytah M: carried out the manuscript editing and manuscript review.

Hopper C: carried out the manuscript editing and manuscript review.

Searle A: carried out the manuscript editing and manuscript review.

Wright A: designed the study, carried out the literature research, clinical study and manuscript preparation.

All authors read and approved the final manuscript.

## Pre-publication history

The pre-publication history for this paper can be accessed here:


